# The role of estrogen deprivation therapy in premenopausal women with primary unresectable intracardiac leiomyomatosis: a systematic review and meta-analysis

**DOI:** 10.1186/s13023-021-02087-7

**Published:** 2021-10-29

**Authors:** Jinxiao Liang, Ruilin Lei, Mingwei Xie, Shaodan Lin, Jing Xu, Xiaoting Ling, Qingsheng Xie

**Affiliations:** 1 Department of Gynecological Oncology, Sun Yat-Sen Memorial Hospital, Sun Yat-Sen University, 107 Yan Jiang Rd West, Guangzhou, 510120 People’s Republic of China; 2 Department of Radiology, Sun Yat-Sen Memorial Hospital, Sun Yat-Sen University, 107 Yan Jiang Rd West, Guangzhou, 510120 People’s Republic of China

**Keywords:** Intracardiac leiomyomatosis, Estrogen deprivation therapy, Primary treatment, Bilateral salpingo-oophorectomy, Gonadotrophin releasing hormone agonists

## Abstract

**Background:**

Intracardiac leiomyomatosis (ICLM) is a rare life-threatening form of intravenous leiomyomatosis (IVLM). The incomplete resection and recurrence are associated with high morbidity and mortality. The objective of this study is to identify that whether estrogen deprivation therapies, including bilateral salpingo-oophorectomy (BSO)-based surgery and gonadotrophin releasing hormone agonists (GnRHa) administration, could bring benefits to patients with primary unresectable ICLM.

**Methods:**

PubMed/MEDLINE (Ovid) was searched (up to May 2021) for studies reporting individual patient data on demographics, clinicopathological features, treatment, and follow-up information. Exclusion criteria were patients who may have been included in two or more publications. This study was performed in accordance with the Preferred Reporting Items for Systematic Reviews and Meta-Analyses (PRISMA) guidelines.

**Results:**

A total of 114 patients from 70 papers were included. Several reports showed that the tumor in the right atrium and inferior vena cava shrank dramatically after BSO-based surgery, or GnRHa administrated preoperatively in premenopausal women. The rate of complete resection was 64.04% in patients with ICLM, which was 85.25% in no/slight adhesion and no pulmonary nodules group, while 22.22% in firm/extensive adhesion and/or pulmonary nodules group (*p* < 0.0001). Meanwhile, the recurrence rates in patients with complete resection and incomplete resection were 4.29% and 37.84% respectively (*p* < 0.0001). Furthermore, complete resection with BSO had the lowest recurrence rate of 3.13%, incomplete resection with BSO had a progression rate of 45.45%, while incomplete resection with ovarian preservation had the highest progression rate of 75.00%.

**Conclusions:**

The recurrence rate of ICLM was closely related to firm/extensive adhesion in IVC or above, and/or pulmonary nodules. BSO-based surgery might reduce the recurrence rate no matter ICLM could be completely resected or not. In addition, estrogen deprivation therapies could decrease tumor burden as a primary treatment, and further make a secondary complete resection feasible in premenopausal women with initially unresectable ICLM.

**Supplementary Information:**

The online version contains supplementary material available at 10.1186/s13023-021-02087-7.

## Background

Intravenous leiomyomatosis (IVLM) is an uncommon smooth muscle tumor that arises from either a uterine-related myoma or the intrauterine vessel walls and extends into the veins [1, 2]. Under a rare condition, it reaches the right atrium by an extensive spread beyond the inferior vena cava (IVC), which is known as intracardiac leiomyomatosis (ICLM) [3]. ICLM is histologically benign but clinically aggressive, which can cause fatalities by acute heart or other organs’ failure [3–6], surgical complications such as uncontrolled hemorrhage [7, 8], and recurrence [9–11].

Treatment for ICLM varied because of its rarity and complexity, which included surgeries [12–14], anti-estrogen hormone therapies [9, 12, 15], and even a wait-and-see strategy [1, 16]. Complete tumor resection was the most recommended [17, 18], but sometimes it cannot be achieved due to the high risks of surgery [5, 7, 13, 15, 16, 19–22], patient’s poor conditions [4, 12, 23, 24], lack of medical resources [25], financial constraints [2, 26], or patients’ refusal [27, 28].

Since ICLM is believed to be estrogen-dependent [1, 2], if the primary complete removal cannot be achieved, could the estrogen deprivation therapy be an initial choice? And could it facilitate a secondary complete resection? Therefore, a systematic literature review and meta-analysis of published patient data was conducted to investigate the feasibility of estrogen deprivation therapies, such as BSO-based surgery and GnRHa administration as the initial treatment of ICLM.

## Methods

### Search strategy

A literature search was performed through PubMed/MEDLINE (Ovid) up to May 2021. The following search terms were used: “intracardiac leiomyomatosis”, “leiomyomatosis extending to right heart” and “leiomyomatosis with cardiac extension”. Two independent investigators screened all studies according to the inclusion and exclusion criteria. In case of disagreement, consensus was met by the senior researcher (Additional file 1: Figure S1). Other publications were found through reference lists of reviewed articles. All the enrolled articles were written in English title and abstract.

### Study selection and data extraction

Studies were eligible if they provided individual patient data on demographics, clinicopathological features, treatment, and follow-up information. Patients were excluded from meta-analysis if any of the following criteria were met: (1) articles written not in English, (2) no specification of treatments, (3) no specification of follow-up data, and (4) pathology findings shown malignant results such as intravenous low-grade endometrial stromal sarcoma. Included case reports and case series were evaluated to assess whether clinicopathological features were provided, specified whether the intravenous leiomyomatosis involved heart. Data of demographics, clinicopathological features, treatment, and follow-up information were extracted by our two independent researchers following the predefined spreadsheet on individual patient level.

### Statistical analysis and quality assessment

All the statistical analysis was performed using SPSS software, version 13.0 (SPSS Inc., Chicago, IL, USA). Associations between the clinicopathological features and the outcomes were examined using chi-square or Fisher’s exact test. All tests were 2-tailed with *p* values < 0.05 considered significant.

Murad et al. provided an approach to evaluate the quality of case reports and case series based on four components: selection, ascertainment, causality and reporting, resulting in a score with a maximum of 5 for both case series and case reports (Additional file 2: Table S1) [29]. There were 2 and 3 of the 8 binary questions not suitable for the case series and case reports, respectively. Quality assessment were evaluated by two independent investigators.

## Results

### Systematic search and study quality

Database search and reference lists identified 241 publications after removal of duplicates (Additional file 1: Figure S1). 109 publications were excluded after screening the titles and abstracts. 132 articles were reviewed and from which 62 were excluded following the inclusion and exclusion criteria. Finally, 70 studies with a total of 114 cases met these criteria in our meta-analysis (Additional file 2: Table S2). 12 studies were case series and 58 studies were case reports, which were published between 1975 and 2021.

Quality assessment identified acceptable study quality with 12 (100%) case series and 48 (82.76%) case reports scoring 4 points or higher in the modified quality evaluation by Murad et al. (Additional file 2: Table S1) [29].

### Clinicopathological features

The clinicopathological features were showed in Table 1. The median age was 46.5 years (range: 24–72 years). The gynecological symptoms that were reported included pelvic pain, menorrhagia, and abdominal discomfort. The cardiopulmonary symptoms that were reported included syncope, dyspnea, chest distress, and lower-extremity edema. The BSO rate in patients admitted by the Department of Obstetrics and Gynecology was 93.33% (28/30), while the BSO rate in patients admitted by Department of Cardio-Thoracic Surgery or other departments was much lower (65.31%, 32/49, *p* = 0.0059). In addition, death associated with ICLM was reported in 3 patients. One died immediately after being admitted to the hospital without surgery [3], one died during surgery [7], and one died of multiple organ failure secondary to tumor progression 11 years after her diagnosis [5]. No leiomyoma embolizing the lung during or after surgery as a tumor thrombosis was reported. 3 patients with atypical histologic features for their ICLM could not achieve complete resection and two of them had a tumor regrowth postoperatively [1, 19, 24].

**Table 1:** Demographic, clinical and treatment factors of 114 reviewed cases of intracardiac leiomyomatosis

Item	Case	
	Number	Percent (%)
**Total number of cases**	**114**	
**Median age in years (range)**	**46.5 (24–72)**	
**Publication Year**	**114**	
Within last 10 years	72	63.16
Earlier than 10 years ago	42	36.84
**Presenting symptoms** ^1^	**102**	
Gynecological symptoms	15	14.71
Cardiopulmonary symptoms	56	54.90
Both gynecological and cardiopulmonary symptoms	12	11.76
Without symptoms	19	18.63
**Menopausal status**	**114**	
Premenopausal or younger than 50 years old	85	74.56
Postmenopausal or older than 50 years old	29	25.44
**Pathological features**	**114**	
Benign leiomyoma	107	93.86
Atypical histologic features	3	2.63
Unknown	4	3.51
**Treatment**	**114**	
Incomplete excision	37	32.46
Complete excision	73	64.04
Unknown	2	1.75
Died perioperatively ^2^	2	1.75
**Admitted by which departments**^1^	**94**	
Department of Cardio-Thoracic Surgery	58	61.70
Department of Obstetrics and Gynaecology	32	34.04
Other Departments ^3^	4	4.26
**Records with BSO or Ovarian Preservation**^1^	**79**	
**Admitted by Department of Obstetrics and Gynaecology**	**30**	
With BSO	28	93.33
Ovarian Preservation	2	6.67
**Admitted by Department of Cardio-Thoracic Surgery or other Departments**	**49**	
With BSO	32	65.31
Ovarian Preservation	17	34.69
**Status of Adhesion at the level of IVC or above (ALIA), Status of pulmonary nodules (PN)**	**114**	
Firm/extensive ALIA, and/or PN	27	23.68
No/slight ALIA, no PN	61	53.51
Unknown	26	21.81

The significance of bold highlight the number of cases in each group or subgroup

^1^available data less than 114; ^2^one died preoperatively, one died postoperatively; ^3^three were admitted by Emergency Department and one were admitted by Department of Gastrointestinal Surgery

### Adhesion situation, pulmonary involvement, and complete resection

The total incomplete resection rate was 32.46% (37 out of 114). Among 114 patients, 27 (23.68%) were reported to have firm/extensive adhesion in IVC or above, and/or had lung metastasis, while 61 (53.51%) had no or only slight adhesion in IVC or above without pulmonary nodules. 26 (21.81%) reported no details. As shown in Table 2, the complete resection rate in no/slight adhesion group was significantly higher than that in firm/extensive adhesion group (*p* < 0.0001). The gentle downward extraction via venotomy of the iliac /IVC veins even without sternotomy could be performed successfully in patients had no/slight adhesion and had no pulmonary nodules [30, 31]. In contrast, the intracaval tumor of some patients were failed to be pulled out due to firmly adhesion [7, 14, 19, 20]. And torn traction in the IVC occurred in one of them, leading to severe bleeding and death during operation [7].

**Table 2:** The complete resection rates between two groups with different adhesion situation and pulmonary involvement

	Complete resection	Incomplete resection	*P*^*#*^
No/slight adhesion and no pulmonary nodules	52 (85.25%)	9 (14.75%)	< 0.0001
Firm adhesion and/or pulmonary nodules	6 (22.22%)	21 (77.78%)	

Valid patient’s numbers: 88; ^#^The P-value was determined using the chi-square test

### Surgical procedure and prognosis

The details of surgical process and follow-ups were reported in 107 patients. The mean follow-up interval was 30.55 months. Totally 17 (15.89%) women underwent recurrence or regrowth of the residual tumor. More recurrence or regrowth of the residual tumors was found in patients with incomplete resections and/or unresectable pulmonary nodules (median follow-up: 26.27 months, range from 2 to 120 months) than in those with complete resection (median follow-up: 32.73 months, range from 1 to 168 months) (37.84% vs. 4.29%, *P* < 0.0001, Table 3). Furthermore, there were 26 women reported with a follow-up interval more than 48 months (48–144 months), and the recurrence rates were 60.00% (6 out of 10) and 12.50% (2 out of 16) between incomplete-resection and complete-resection patients (*p* = 0.0256, Additional file 2: Table S4), respectively.

**Table 3:** The rates of residual tumor regrowth between two groups with complete or incomplete resection

	Recurrence or regrowth (n = 14)	No progression (n = 68)	*P*^*#*^
Complete resection	3 (4.29%)	67 (95.71%)	< 0.0001
Incomplete resection	14 (37.84%)	23 (62.16%)	

Valid patient’s numbers: 107; ^#^The *P*-value was determined using the chi-square test

Among these 107 patients, reports on whether BSO was performed or not was found in 71 women who were premenopausal or younger than 50 years old (Table 4). Data demonstrated that patients with complete resection and BSO had the best prognosis, only 3.13% occurred with tumor recurrence after surgery. Incomplete tumor resection with BSO had a progression rate of 45.45%, while patients with incomplete resection with ovarian preservation had the highest rate of residual tumor regrowth (75.00%). However, if complete tumor resection can be achieved, patients with ovarian preservation still had an acceptable recurrence rate of 15.38%.

**Table 4:** Different treatments and residual tumor progression in premenopausal women with ICLM

Complete resection (13)		Incomplete resection (4)	
Progression	No progression	Progression	No progression
*Ovarian Preservation (17)*			
2 (15.38%)	11 (84.62%)	3 (75.00%)	1 (25.00%)

Complete resection (32)		Incomplete resection (22)	
Progression	No progression	Progression	No progression
BSO (54)			
1 (3.13%)	31 (96.88%)	10 (45.45%)	12 (54.55%)

Valid patient’s numbers: 71; BSO: Bilateral salpingo-oophorectomy

### Pre/postoperative GnRHa and ICLM

GnRHa is extremely efficient and widely used to reduce tumor volume and reverse the related symptomatology for premenopausal women with uterine fibroids [32]. However, the tumors tend to return to their pretreatment size around 6 months after discontinuing treatment. And this phenomenon was also observed in our study. There were 8 women who received GnRHa pre or postoperatively and in the details were reported (Additional file 2: Table S5). All of them were premenopausal, either had no complete resection or later emerged with recurrence. The residual tumor was partly reduced in 3 patients with preoperative GnRHa administration initially [19, 33, 34], but the effects were limited and the tumor increased in size later soon. 3 patients had ovarian preservation and administration of GnRHa postoperatively (15 to 24 months), and the tumors were effectively suppressed or disappeared during the treatment [9, 15, 35]. The other 2 patients had received incomplete resection with BSO and the administration of GnRHa postoperatively, the residual tumors remained stable [36, 37].

## Discussion

ICLM is a rather rare pathologic condition and usually occurs among premenopausal women with a history of uterine leiomyoma. All the patients in this study were treated in enhanced or maximal-resource countries or areas. Patients tended to be well-treated, which at least partly lead to survivorship bias. Consequently, the incidence and the recurrence rate, the morbidity, and mortality in the whole world could be higher [38]. Moreover, it still remains controversial on the treatment strategy for the patients with ICLM, especially for those in developing areas or with less medical insurance, who may face unaffordable medical expenses and the shortage of medical resources [2, 26, 39]. Therefore, Li et al. emphasized that less invasive surgery without compromising the curative effect would be the main goal [39].

Complete removal of the ICLM plus BSO was the most optimal and efficient treatment in preventing recurrences (Table 4), which was consistent with other studies [17, 18, 23, 38, 40]. And the patients with complete-resection had a pronouncedly lower rate of recurrence when compared with that in those with incomplete-resection (Table 3).

However, complete resection cannot be performed in many cases for extension of tumors [5, 24, 27, 37], densely adherent to the luminal wall [4, 5, 7, 13–16, 19–22, 41, 42], and patients’ will [27, 28], or constrained medical resources. In such conditions, recurrence occurred postoperatively [4, 9, 10, 12, 15, 21, 22, 24], lethal complications such as uncontrolled hemorrhage [20, 42], heart failure, and sudden death happened [3, 7]. The complete resection rate was significantly lower in patients with firm/extensive adhesion than that in those with no/slight adhesion of IVC or above (Table 2). Among patients with incomplete resection, 37.84% had recurrence, which was significantly higher when compared with 4.29% in those with complete resection (Table 3). Correspondingly, with a longer follow-up interval, the recurrence rate was dramatically higher of 60.00% and 12.50% respectively (Additional file 2: Table S4). Therefore, it is a dilemma whether to suspend a high-risk surgery which may lead to incomplete resection and recurrence, or perform a complete resection which might be life-threatening.

Despite limited data, our research showed that the 15 patients tested by IHC were all positive for ER and/or PR (Additional file 2: Table S3), which means it could be useful for the patients with ICLM to be treated with hormone-based therapies. Consistently, a premenopausal case showed that incomplete resection but BSO-based surgery could render the residual tumor reduced significantly postoperatively [34]. In another case, the unresectable IVLM shrank significantly by 9 months of GnRHa therapy, which could be completely removal thereafter [43]. GnRHa given pre or postoperatively in premenopausal women was shown to be able to reduce preoperative tumors size, and suppress the progression of the residual tumor [9, 15, 19, 33, 34, 36, 37]. Moreover, the intraoperative blood loss and medical costs was 7.5 and 7 times higher in patients with ICLM when compared with patients with IVLM but not extended beyond the abdominal cavity [18]. So, the application of estrogen deprivation therapies before the surgery might efficiently reduce the perioperative complications, and could further facilitate a secondary complete resection in premenopausal women. On the other hand, if the complete tumor resection cannot be achieved, ovarian preservation could lead to a notably high recurrence rate of 75.00% (Table 4). Therefore, whether complete resection could be accomplished or not, the estrogen deprivation therapies would be beneficial for treating the ICLM, especially when which was highly risky for completely resection, in the premenopausal women (Table 4, Additional file 2: Table S5).

One other point worth emphasizing was that, patients admitted by the department of Thoracic and Cardiovascular Surgery or other departments had a significantly lower rate of BSO when compared to those patients admitted by the department of Obstetrics and Gynecology (Table 1). Considering the benefit brought by the BSO, gynecologists should be involved in the treatment decision-making process of ICLM.

However, this meta-analysis has several limitations because all studies analyzed were observational with risks of bias. Potential bias was attempted to be minimized by excluding studies which failed to provide patient characteristics and follow-up information. The effects of estrogen deprivation therapies for ICLM were observed in not long follow-up intervals, and the data size was small due to the scarcity of this disease. Moreover, though most of the ICLM was found to have positive expression of estrogen receptor, antiestrogen therapy might be less beneficial in postmenopausal than in premenopausal women [44]. Further studies are needed to evaluate the rate of secondary complete resection, the long-term effects of estrogen deprivation therapy and the differences between the premenopausal women and the postmenopausal women.

## Conclusions

Estrogen deprivation therapies may be essential in treating primary unresectable ICLM for its effects in shrinking the tumor size, suppressing the tumor progression, and facilitating a secondary complete resection. In other words, estrogen deprivation therapies could render a less invasive secondary surgery but without compromising the curative effect.

## Supplementary Information

**Additional file 1: Figure S1**: PRISMA flow diagram outlining study selection. Data searches were performed up to June 2021.

**Additional file 2**: Supplemental material to support the methods and results.

## Data Availability

Datasets supporting conclusions of this article are included within the article.

## Abbreviations

ICLM: Intracardiac leiomyomatosis
IVLM: Intravenous leiomyomatosis
BSO: Bilateral salpingo-oophorectomy
GnRHa: Gonadotrophin releasing hormone agonists
PRISMA: Preferred reporting items for systematic reviews and meta-analyses
IVC: Inferior vena cava
IHC: Immunohistochemistry
ER: Estrogen receptor
PR: Progesterone receptor
