# Supplementary figures and images for: The role of estrogen deprivation therapy in premenopausal women with primary unresectable intracardiac leiomyomatosis: a systematic review and meta-analysis
Source: Orphanet J Rare Dis. 2021 Oct 29;16:453. doi: 10.1186/s13023-021-02087-7 (PMC8555203; doi:10.1186/s13023-021-02087-7)

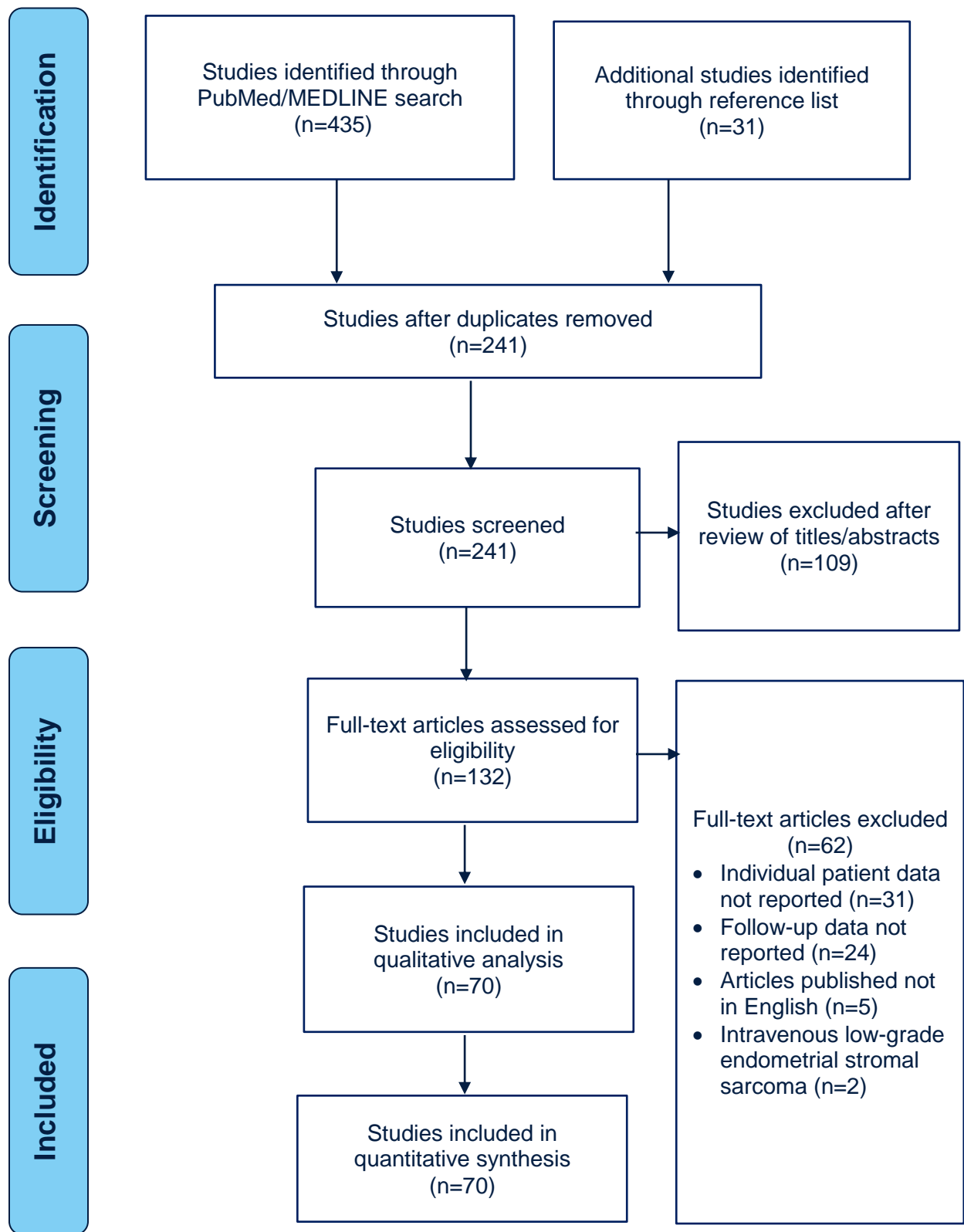

Supplement: Supplementary file 1 — Additional file 1: Figure S1: PRISMA flow diagram outlining study selection. Data searches were performed up to June 2021. [file 13023_2021_2087_MOESM1_ESM.pdf]
