# Supplementary material for: The role of estrogen deprivation therapy in premenopausal women with primary unresectable intracardiac leiomyomatosis: a systematic review and meta-analysis
Source: Orphanet J Rare Dis. 2021 Oct 29;16:453. doi: 10.1186/s13023-021-02087-7 (PMC8555203; doi:10.1186/s13023-021-02087-7)
Supplement: Supplementary file 2 — Additional file 2: Supplemental material to support the methods and results. [file 13023_2021_2087_MOESM2_ESM.docx]

**Suppl. Table 1.** The quality assessment summary of included studies

| **Type** | **Number of studies** | **Year** | **Score** |
| --- | --- | --- | --- |
| Case series | 9 | 1975-2021 | 5 |
|  | 3 | 2011-2015 | 4 |
| Case report | 3 | 1983-2003 | 5 |
|  | 45 | 1982-2021 | 4 |
|  | 9 | 1980-2017 | 3 |
|  | 1 | 1998 | 2 |

**Suppl. Table 2.** Studies included in the meta-analysis

Li M, Guo C, Lyu YH, Zhang MB, Wang ZL. An unusual case of intravenous leiomyomatosis involving the right atrium. Chin Med J. 2019; 132:474-6.

Osawa H, Hosaka S, Akashi O, Furukawa H, Egi K. A case of intravenous leiomyomatosis of uterine origin, extending through the inferior vena cava to right atrium. Gen Thorac Cardiovasc Surg 2013; 61:104-7.

Liu W, Liu M, Xue J. Detection of intravenous leiomyomatosis with intracardiac extension by ultrasonography: A case report. Oncol Lett 2013; 6: 336-8.

Rosenberg JM, Marvasti MA, Obeid A, Johnson LW, Bonaventura M. Intravenous leiomyomatosis: a rare cause of right sided cardiac obstruction. Em J Cardio-thorac Surg 1988; 2:58-60.

Du J, Zhao X, Guo D , Li H, Sun B. Intravenous leiomyomatosis of the uterus A clinicopathologic study of 18 cases, with emphasis on early diagnosis and appropriate treatment strategies. Hum Pathol 2011; 42:1240-6.

Iverson LIG, Lee J, Drew D, Sharp J, Ecker RR, Young JN, et al. Intravenous Leiomyomatosis with Cardiac Extension. Tex Heart Inst J 1983; 275-8.

Daphna MD, Pitlik S, Sagie A, Ovadia Y, Bishara J. Intravenous leiomyomatosis with cardiac involvement in a pregnant woman. Am J Obstet Gynecol 1998; 620-1.

Khayata GM, Thwaini S, Aswad SG. Intravenous leiomyomatosis extending to the heart. Int J Gynaecol Obstet2003; 80:59–60.

Morice P, Chapelier A, Dartevelle P, Castaigne D, Lhomme C. Late Intracaval and Intracardiac Leiomyomatosis Following Hysterectomy for Benign Myomas Treated by Surgery and GnRH Agonist. Gynecol Oncol 2001; 83:422-3.

Gehr NR, Lund O, Alstrup P, Nielsen JS, Villadsen AB, Bartholdy NJ. Recurrence of Uterine Intravenous Leiomyomatosis with Intracardiac Extension. Scand Cardiovasc J 1999; 33; 312-4.

Worley MJ Jr, Aelion A, Caputo TA, Kent KC, Salemi A, Krieger KH, et al. Intravenous leiomyomatosis with intracardiac extension: a single-institution experience. Am J Obstet Gynecol 2009; 201: 574. e1-5.

To WWK, Ngana HYS, Collinsb RJ. Intravenous leiomyomatosis with intracardiac involvement. Int J Gynecol Obstet 1993, 42: 37-40.

Castelli P, Caronno R, Piffaretti G, Tozzi M. Intravenous Uterine Leiomyomatosis with Right Heart Extension: Successful Two-Stage Surgical Removal. Ann Vasc Surg 2006; 20: 405-7.

Yu HY, Tsai HE, Chi NH, Kuo KT, Wang SS, Chen CA, et al. Long-term outcomes of surgical treatment for intravascular leiomyomatosis. J Formos Med Assoc 2018; 117:964-72.

Konishi H, Koh I, Shiroma N, Kidani Y, Urabe S, Tanaka N, et al. Two Case Reports of Intravenous Leiomyomatosis with Hyaluronan Expression. Case Reports in Obstetrics and Gynecology 2018; DOI: https://doi.org/10.1155/2018/4039183.

Nakai G, Maeda K, Yamamoto K, Yamada T, Hirose Y, Terai Y, et al. Uterine Intravenous Leiomyomatosis with Cardiac Extension: Radiologic Assessment with Surgical and Pathologic Correlation. Case Reports in Obstetrics and Gynecology 2015; DOI: http://dx.doi.org/10.1155/2015/576743.

Lian C, Yin S, Qiu J, Cui J, Hu Z, Chang G, et al. Experience in the diagnosis and treatment of intravenous leiomyomatosis involving the inferior vena cava and/or right cardiac chambers. J Vasc Surg Venous Lymphat Disord 2020; DOI: <https://doi.org/10.1016/j.jvsv.2020.05.011>.

Liu N, Long Y, Liu Y. Intravenous leiomyomatosis: Case series and review of the literature. J Int Med Res 2020; 48:1-7.

Kaur S, Tongaonkar HB, Maheshwari A, Menon S. A rare case of recurrent intravenous leiomyomatosis: Role of GnRH analogues? Indian J Cancer 2019; 52:161.

Shi T, Shkrum M. A Case Report of Sudden Death From Intracardiac Leiomyomatosis. Am J Forensic Med Pathol 2018; DOI: 10.1097/PAF.0000000000000377.

Kim JH, Baek JH, A challenging case of intracardiac leiomyomatosis accompanied by Pseudo-Meigs syndrome originating from uterine leiomyoma, Ann Vasc Surg 2018; DOI: 10.1016/j.avsg.2018.06.026.

Schäfer HM, Isaak A, Gürke L. Case report of an intracaval leiomyomatosis 10 months after complete hysterectomy. Int J Surg Case Rep 2017; 35:1-3.

Rispoli P, Santovito D, Tallia C, Varetto G, Conforti M, Rinaldi M, et al. A one-stage approach to the treatment of intravenous leiomyomatosis extending to the right heart. J Vasc Surg 2010; 52:212-5.

Anselmi A, Tsiopoulos V, Perri G, Palladino M, Ferrante A, Gliec F. Case series of resection of pelvic leiomyoma extending into the right heart: surgical safeguards and clinical follow-up. J Cardiovasc Med (Hagerstown) 2010, 11:583-6.

Uchida H, Hattori Y, Nakada K, Iida D. Successful One-Stage Radical Removal of Intravenous Leiomyomatosis Extending to the Right Ventricle. Obstet Gynecol 2004; 103: 1068-70.

Lam PM, Lo KWK, Yu MMY, Lau TK, Cheung TH. Intravenous leiomyomatosis with atypical histologic features: A case report. Int J Gynecol Cancer 2003; 13, 83-7.

Gawne-Cain ML, Wilson AG, Corbishley C, Keating V, Joseph AEA. (1995). Case Report: Intravenous Leiomyomatosis, an Unusual Cause of Intracardiac Filling Defect. Clin Radiol 1995; 50:123-5.

Ricci MA, Cloutier LM, Mount S, Welander C, Leavitt BJ. Intravenous Leiomyomatosis with Intracardiac Extension. Cardiovascular Surgery 1995; 3: 693-6.

Suginami H, Kaura R, Ochi H, Matsuura S. Intravenous Leiomyomatosis with Cardiac Extension: Successful Surgical Management and Histopathologic Study. Obstet Gynecol 1990; 76: 527-9.

Nili M, Liban M, Levy MJ. Tricuspid Stenosis due to Intravenous Leiomyomatosis - A Call for Caution: Case Report and Review of the Literature. Tex Heart Inst J 1982; 231-5.

Norris HJ, Parml T. Mesenchymal Tumors of the Uterus. V. Intravenous Leiomyomatosis: A Clinical and Pathologic Study of 14 Cases. Cancer 1975; 36: 2164-78.

Mitsuhashi A, Nagai Y, Sugita M, Nakajima N, Sekiya S. GnRH Agonist for Intravenous Leiomyomatosis with Cardiac Extension: A Case Report. J Reprod Med 1999; 44: 883-6.

Doganci S, Kaya E, Kadan M, Karabacak K, Erol G, Demirkilic U. Huge Intravascular Tumor Extending to the Heart: Leiomyomatosis. Case Reports in Surgery 2015; DOI: <http://dx.doi.org/10.1155/2015/658728>.

Mizuno T, Mihara A, Arai H. Intracardiac and intravascular leiomyomatosis associated with a pelvic arterio-venous fistula. Ann Transl Med 2014; DOI: 10.3978/j.issn.2305-5839.2014.04.14.

Kullo IJ, Oh JK, Keeney GL, Khandheria BK, Seward JB. Intracardiac Leiomyomatosis: Echocardiographic Features. Chest 1999; 115:587-91.

Lo KWK, Lau TK. Intracardiac leiomyomatosis: Case report and literature review. Arch Gynecol Obstet 2001; 264:209-10.

Timmis AD, Smallpeice C, Davies AC, Gishen P, Jackson G. Intracardiac Spread of Intravenous Leiomyomatosis with Successful Surgical Excision. N Engl J Med 1980; 303: 1043-4.

Barksdale J, Abolhoda A, Saremi F. Intravenous leiomyomatosis presenting as acute Budd-Chiari syndrome. J Vasc Surg 2011; 54: 860-3.

Harris LM, Karakousis CP. Intravenous leiomyomatosis with cardiac extension: Tumor thrombectomy through an abdominal approach. J Vasc Surg 2000; 31:1046-51.

Ayling O, Roy T, Cusimano RJ, McGilvray I, Roche-Nagle G. Intravenous leiomyomatosis with cardiac extension. Vasa 2015; 44: 151-5.

Saitoh M, Hayasaka T, Nakahara K, Ohmichi M, Shimazaki Y, Kurachi H. Intravenous Leiomyomatosis with Cardiac Extension. Gynecol Obstet Invest 2004; 58: 168-70.

He J, Chen ZB, Wang SM, Liu MB, Li ZG, Li HY, et al. Intravenous leiomyomatosis with different surgical approaches: Three case reports. World J Clin Cases 2019; 7: 347-56.

Fang BR, Ng YT, Yeh CH. Intravenous Leiomyomatosis With Extension to the Heart: Echocardiographic Features: A Case Report. Angiology. 2007; 58: 376-9.

Liu B, Liu C, Guan H, Li Y, Song X, Shen K. Intravenous leiomyomatosis with inferior vena cava and heart extension. J Vasc Surg 2009; 50: 897-902.

Zhang G, Yu X, Lang J. Intravenous leiomyomatosis with inferior vena cava or intracardiac extension and concurrent bilateral multiple pulmonary nodules: A report of 2 cases. Medicine 2016; 95: 35 (e4722).

Clay TD, Dimitriou J, McNally OM, Russell PA, Newcomb NE, Wilson AM. Intravenous leiomyomatosis with intracardiac extension e A review of diagnosis and management with an illustrative case. Surg Oncol 2013; 22: e44-52.

Lee PK, David TE, Sloggett C, Ross JR. Intravenous leiomyomatosis with intracardiac extension: an unusual cause of cardiac syncope. Can Med Assoc J 1990; 1257-9.

Gissey LC, Mariano G, Musleh L, Lepiane P, Colasanti M, Meniconi RL, et al. Massive pelvic recurrence of uterine leiomyomatosis with intracaval-intracardiac extension: video case report and literature review. BMC Surgery 2017; 17: 118.

Marrone G, Crinò F, Morsolini M, Caruso S, Miraglia R. Multidisciplinary approach in the management of uterine intravenous leiomyomatosis with intracardiac extension: case report and review of literature. Radiology Case 2019; 13:1-13.

Zhang Y, Zhu J, Wang C, Tu R, Jiang J, Lu W. Multimodality treatment of two cases of intracardiac leiomyomatosis with enormous mass in the abdominopelvic cavity. Expert Rev Anticancer Ther 2013; 13: 137-41.

Wu YH, Lee YT, Lee C, Tzeng YH, Wei J. Nonthrombotic pulmonary embolism caused by intravenous leiomyomatosis: A case report. Medicine 2019; 98:3.

Okamura H, Yamaguchi A, Kimura N, Adachi K, Adachi H. Partial resection of intravenous leiomyomatosis with cardiac extension. Gen Thorac Cardiovasc Surg 2011; 59: 38-41.

Nam MS, Jeon MJ, Kim YT, Kim JW, Park KH, Hong YS. Pelvic leiomyomatosis with intracaval and intracardiac extension: a case report and review of the literature. Gynecol Oncol 2003; 175-80.

Kokawa K, Yamoto M, Yata C, Mabuchi Y, Umesaki N. Postmenopausal Intravenous Leiomyomatosis With High Levels of Estradiol and Estrogen Receptor. Obstet Gynecol 2002; 100: 1124-6.

Pesi B, Moraldi L, Antonuzzo L, Meoni G, Addasi R, Montesi G, et al. Single-stage operation using hypothermic circulatory arrest to remove uterine intravenous leiomyomatosis extended to the vena cava and right atrium. Int J Gynaecol Obstet 2015; 129: 87-8.

Arinami Y, Kodama S, Kase H, Tanaka K, Okazaki H, Maruyama Y. Successful One-Stage Complete Removal of an Entire Intravenous Leiomyomatosis in the Heart, Vena Cava, and Uterus. Gynecol Oncol 1997; 64: 547-50.

Fujiwara K, Haba M, Noguchi Y, Yamamoto S, Iwasaki M. Successful One-stage Surgical Removal of Intravenous Uterine Leiomyomatosis with Right Heart Extension. Jpn J Thorac Cardiovasc Surg 2003; 51: 462-5.

Galajda Z, Copotoiu C, Suciu H, Tint D, Glasz T, Deac R. The diagnosis, morphological particularities, and surgical technique in a case of intravascular leiomyoma extended to the right heart chambers. J Vasc Surg 2010; 51: 1000-2.

Doyle MP, Li A, Villanueva CI, Peeceeyen SCS, Cooper MG, Hanel KC, et al. Treatment of Intravenous Leiomyomatosis with Cardiac Extension following Incomplete Resection. Int J Vasc Med 2015; DOI: <https://doi.org/10.1155/2015/756141>.

Sogabe M, Kawahito K, Aizawa K, Sato H, Misawa Y. Uterine Intravenous Leiomyomatosis with Right Ventricular Extension. Ann Thorac Cardiovasc Surg 2014; Supplement: 933-6.

Price JD, Anagnostopoulos C, Benvenisty A, Kothuru RK, Balaram SK. Intracardiac Extension of Intravenous Leiomyomatosis. Ann Thorac Surg 2017; 103: e145-7.

Corbett GA, O’Gorman C, Kamran W. Intravenous leiomyomatosis: the first reported case of intraoperative intracaval embolisation of tumour to the right atrium. BMJ Case Rep 2020; 13: e233341. doi:10.1136/bcr-2019- 233341.

Zhang G; Feng F; Wang W; Zhu L. Rapamycin (Sirolimus) in treatment of recurrent intravenous leiomyomatosis: a case report. BJOG 2020; 127: 768-71.

Yano M, Katoh T, Nakajima Y, Iwanaga S, Kin R, Kozawa E. Uterine intravenous leiomyomatosis with an isolated large metastasis to the right atrium: a case report. Diagn Pathol 2020; 15:4.

Li H, Xu D, Lu W, Wang C. Complete resection of intracardiac leiomyomatosis through an abdominal approach under peripheral cardiopulmonary bypass. J Thorac Cardiovasc Surg 2016;152: e91-3.

Li H, Xu J, Lin Q, Zhang Y, Zhao Y, Tong H, et al. Surgical treatment strategies for extra-pelvic intravenous leiomyomatosis. Orphanet J Rare Dis. 2020; 15:153. doi: 10.1186/s13023-020-01394-9.

Park S, Yeo IH, Kim YJ, Kim JK. Obstruction of the Hepatic Venous Flow Caused by Intravenous Leiomyomatosis. Medicina (Kaunas). 2020; 56(12): 696. doi: 10.3390/medicina56120696.

Judson I, Messiou C. Vitamin D deficiency in the pathogenesis of leiomyoma and intravascular leiomyomatosis: A case report and review of the literature. Gynecol Oncol Rep. 2021; 35: 100681. doi: 10.1016/j.gore.2020.100681.

Deng Y, Song B. Three Case Reports of Intravenous Leiomyomatosis with Intracardiac Extensions. Thorac Cardiovasc Surg Rep. 2020; 9(1): e40-e43. doi:10.1055/s-0040-1715183

**Suppl. Table 3.** IHC analyses of smooth muscle markers, ER and PR expression in 29 patients with ICLM

| NO. | Author (year) | ER | PR | Smooth muscle markers |
| --- | --- | --- | --- | --- |
| 1 | Ricci MA, et al (1995) | NE | NE | SMA, Vimentin |
| 2 | Daphna MD, et al (1998) | NE | NE | Desmin |
| 3 | Kullo IJ, et al (1999) | NE | NE | SMA, Keratin |
| 4 |  | NE | NE | SMA, Keratin |
| 5 | Lam PM, et al (2003) | NE | NE | SMA, Desmin |
| 6 | Saitoh M, et al (2004) | + | + | NE |
| 7 | Castelli P, et al (2006) | NE | NE | Desmin, SMA |
| 8 | Fang BR, et al (2007) | + | NE | NE |
| 9 | Liu B, et al (2009) | +*^#^* | | NE |
| 10 |  | +*^#^* | | NE |
| 11 | Rispoli P, et al (2010) | NE | NE | SMA, Desmin |
| 12 | Galajda Z, et al (2010) | NE | NE | SMA, H-caldesmon |
| 13 | Du J, et al (2011) | NE | NE | SMA |
| 14 | Clay TD, et al (2013) | + | + | SMA |
| 15 | Doyle MP, et al (2015) | + | + | SMA, Desmin |
| 16 |  | + | + | SMA, Desmin |
| 17 | Zhang G, et al (2016) | + | + | SMA, Desmin |
| 18 |  | + | + | SMA, Desmin |
| 19 | Schäfer HM, et al (2017) | NE | NE | SMA, Desmin |
| 20 | Gissey LC, et al (2017) | NE | NE | SMA, Desmin |
| 21 | Yu HY, et al (2018) | NE | NE | SMA, Desmin |
| 22 |  | NE | NE | SMA, Desmin |
| 23 |  | NE | NE | SMA, Desmin |
| 24 | Li M, et al (2019) | + | + | Desmin, Vimentin, SMA |
| 25 | He J, et al (2019) | + | + | Caldesmon, SMA |
| 26 | Zhang G, et al (2020) | + | + | SMA, Desmin |
| 27 | Yano M, et al (2020) | + | - | SMA, Desmin |
| 28 | Park S, et al (2020) | + | + | NE |
| 29 | Liang J, et al (2021) | + | + | HHF35, Desmin |

*IHC was performed only in 25.4% (29 out of 114) of patients. NE: not evaluated; ^#^ the two patients were positive at least for one marker of ER and PR.*

**Suppl. Table 4.** The residual tumor regrowth rate between two groups with complete or incomplete resection with longer follow-up interval

| \|  \| **Recurrence or regrowth (n=6)** \| **No progression (n=12)** \| ***P^#^*** \| \| --- \| --- \| --- \| --- \| \| Complete resection \| 2 (12.50%) \| 14 (87.50%) \| 0.0256 \| \| Incomplete resection \| 6 (60.00%) \| 4 (40.00%) \|  \| |
| --- | --- | --- | --- | --- | --- | --- | --- | --- | --- | --- | --- | --- |

*Valid patient’s numbers: 26; Follow-up interval ≥48 months； ^#^The P-value was determined using the chi-square test.*

**Suppl. Table 5.** GnRHa administration, BSO and ICLM with incomplete resection

| **GnRHa Administration Time** | **BSO/ Ovarian preservation** | **Number** | **Effects** |
| --- | --- | --- | --- |
| Pre- Operation | - | 3 | Regressed initially, increased later |
| Post-Operation | BSO | 2 | Residual stable |
| Post-Operation | Ovarian preservation | 3 | Residual inhibited significantly |

*Valid patient’s numbers: 8*
